# Targeting endosomal acidification by chloroquine analogs as a promising strategy for the treatment of emerging viral diseases

**DOI:** 10.1002/prp2.293

**Published:** 2017-01-23

**Authors:** Md. Abdul Alim Al‐Bari

**Affiliations:** ^1^ Department of PharmacyUniversity of RajshahiRajshahi6205Bangladesh

**Keywords:** Chloroquine analogs, antiviral actions, endosomal pH and viral replication

## Abstract

Emerging viruses such as HIV, dengue, influenza A, SARS coronavirus, Ebola, and other viruses pose a significant threat to human health. Majority of these viruses are responsible for the outbreaks of pathogenic lethal infections. To date, there are no effective therapeutic strategies available for the prophylaxis and treatment of these infections. Chloroquine analogs have been used for decades as the primary and most successful drugs against malaria. Concomitant with the emergence of chloroquine‐resistant *Plasmodium* strains and a subsequent decrease in the use as antimalarial drugs, other applications of the analogs have been investigated. Since the analogs have interesting biochemical properties, these drugs are found to be effective against a wide variety of viral infections. As antiviral action, the analogs have been shown to inhibit acidification of endosome during the events of replication and infection. Moreover, immunomodulatory effects of analogs have been beneficial to patients with severe inflammatory complications of several viral diseases. Interestingly, one of the successful targeting strategies is the inhibition of HIV replication by the analogs in vitro which are being tested in several clinical trials. This review focuses on the potentialities of chloroquine analogs for the treatment of endosomal low pH dependent emerging viral diseases.

**Abbreviations**
ASA: acetylsalicylic acid
CQ: chloroquine
DP: dihydroartemisinin‐piperaquine
HCQ: hydroxychloroquine
HCV: hepatitis C virus
HIV: human immunodeficiency virus
IDO: indoleamine 2,3‐dioxygenase
IFN: interferon
MQ: mefloquine
NO: nitric oxide
PML: progressive multifocal leukoencephalopathy
PS: prednisone
ROS: reactive oxygen species
SARS: severe acute respiratory syndrome
SP: sulfadoxine‐Pyrimethamine
TLR: toll‐like receptor
TNF: tumor necrosis factor

## Introduction

Emerging and re‐emerging pathogens such as Ebola and Marburg viruses; dengue and hepatitis C viruses; severe acute respiratory syndrome (SARS) and Middle‐East respiratory syndrome (MERS), coronaviruses; human and avian influenza viruses; Chikungunya virus (CHIKV); human immunodeficiency virus (HIV) and other viruses represent huge challenges to human and veterinary medicine. Researchers, physicians, and healthcare professionals work together to evaluate their pandemic potentials and plan mitigating strategies. For entry into hosts, viruses bind to surface molecules on the plasma membrane of susceptible cells ‐ such as macrophages, monocytes, dendritic cells, endothelial cells, and hepatocytes and lead to them being internalized into vesicles which traffic through the endosomal/lysosomal pathways (Kissing et al. 2014; Shivanna et al. 2014; Bekerman and Einav 2015; Ekins et al. 2015; Kraft et al. 2015; Long et al. 2015). In order to infect susceptible cells, the viruses require endosomal/lysosomal acidification and the acidic pH dependent endosomal proteases cleave the viral glycoprotein segments to cross the replication events (Chandran et al. 2005; Marzi et al. 2012). Without endosomal acidification and cleavage processes, the viral replication and infection are abrogated (Martinson et al. 2014; Shivanna et al. 2014). Therefore, targeting the endosomal/lysosomal acidification and their acidic pH dependent proteases by the therapeutic agents will be highly effective in combating the present world viral epidemic.

Successful viral infection results in the local and systemic releases of several cytokines, chemokines, reactive oxygen species (ROS), nitric oxide (NO) and other mediators (Villinger et al. 1999; Baize et al. 2002) and causes a generalized cell death (Gandini et al. 2013; Meng et al. 2013; Routy et al. 2014). If the immune system of patients is able to control the infection, the patients recover, though convalescence is prolonged and recovered patients have been shown to produce infectious virus up to 3 months after clinical symptoms have disappeared. However, if the immune system of patients is unable to regulate the infection, further susceptible cells are infected, leading to excessive release of the above cytokines. This manifestation is fatal because extensive necrotic cells are present in many organs ‐ including the liver, spleen, lymph nodes, and kidney (Leroy et al. 2000; Baize et al. 2002).

Chloroquine and its structural analogs such as hydroxychloroquine, pamaquine, plasmoquine, primaquine, mefloquine, or ferroquine (ferrocenic analog of chloroquine) have been used for decades as the primary and most successful drugs against malaria. Concomitant with the emergence of chloroquine‐resistant *Plasmodium* strains and a subsequent decrease in the use as antimalarial drugs, new potential uses of the cheap and available analogs have been investigated. Due to their immunomodulatory effects, the analogs have been used as secondary drugs to treat a variety of chronic autoimmune diseases (e.g., rheumatoid arthritis, systemic lupus erythematosus etc.), tumors, and nonmalarial infections (Al‐Bari 2015). Recently, several efforts have been made to identify effective, inexpensive, and universally available antiviral agents. In these senses, the analogs have been suggested as such antiviral agents by inhibiting the replications and infections (Geisbert et al. 2003; Savarino et al. 2003; Barrow et al., 2013).

## Therapeutically Exploitation as Lysosomotropic Property of Chloroquine Analogs

**Figure 1:**
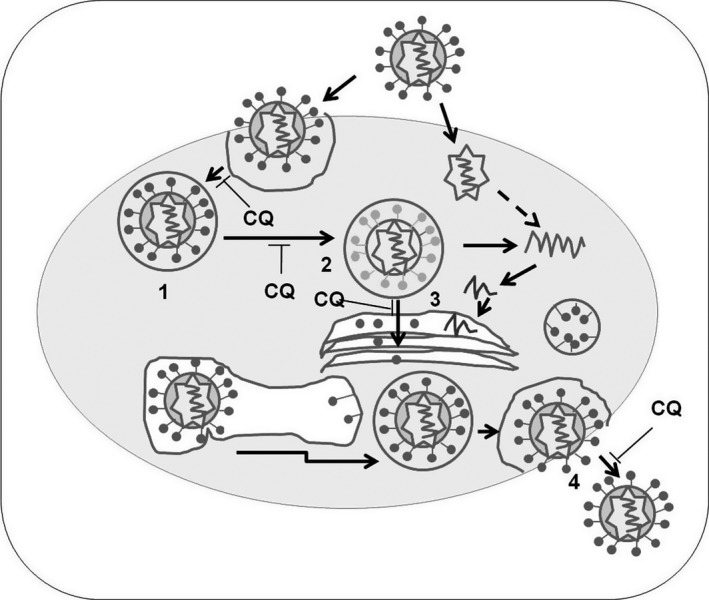
Inhibition of viral infection with the increase pH by chloroquine analogs ((Al‐Bari 2015). Steps: 1. Endosome formation; 2. Fusion; 3. posttranslational modification; 4. uncoating virus and CQ, chloroquine.

Chloroquine analog is a diprotic weak base. The unprotonated form of chloroquine diffuses spontaneously and rapidly across the membranes of cells and organelles to acidic cytoplasmic vesicles such as endosomes, lysosomes, or Golgi vesicles and thereby increases their pH (Al‐Bari 2015). On oral administration, the analog is readily absorbed and concentrated in tissues such as the liver, spleen, and kidney (Al‐Bari 2015)‐ where several fatal viruses harbored, replicated, and infected (Geisbert et al. 2003). In cellular levels of the tissues, chloroquine becomes highly concentrated in such acidic organelles leading to dysfunction of several enzymes, e.g. those required for proteolytic processing and post‐translational modification of viral proteins (Fig. 1) (Savarino et al. 2003; Marzi et al. 2012). Consequently, chloroquine analogs inhibit the production of several cytokines, chemokines or mediators, whose excessive appearance contributes the severity of viral infections. Therefore, the inhibition of endosomal acidification by chloroquine analogs may become a potential therapeutic strategy for viral infections and associated pathologies.

The increasing evidence suggests that the entry, replication and infection processes of several viruses such as Ebola, Marburg, dengue, Chikungunya, HIV etc. are highly dependent on endosomal‐lysosomal acidification and the activities of several host endosomal proteases ‐ which are also active in acidic pH environments (Sun and Tien 2012; Barrow et al. 2013). By neutrality of acidic pH in endosomes, chloroquine analogs inhibit these viral entry and replication processes into the cytoplasm of susceptible cells and thereby abrogate their infections (Chiang et al. 1996; Savarino et al. 2003). Furthermore, the dysfunction of various enzymes e.g. glycosylating enzymes, glycosyltransferases caused by increased acidic pH and/or structural changes in the Golgi apparatus with hydroxychloroquine or by specific interaction with chloroquine, have been shown to suppress not only glycosylation of SARS‐ coronaviruses (Vincent et al. 2005; Savarino et al. 2006) but also that of the HIV‐1 gp120 envelope protein, resulting in structural changes in the gp120 glycoprotein, which in turn reduce the reactivity and infectivity of newly produced virions (Savarino et al. 2004; Naarding et al. 2007). Since the surface glycoproteins of filoviruses (Ebola and Marburg) involve in initiation of infection (Takada et al. 1997; Yang et al. 2000), and cytotoxicity (Yang et al. 2000), the inhibition of glycosylation by the analogs prevents the viral entries for a wide variety of host cells and leads to suppress their pathogenicity by producing of noninfectious or decreased infectivity viruses. This inhibited glycosylation will therefore allow time for the adaptive immune response to deal with the infection (Baize et al. 1999).

## Immunomodulatory Property of Chloroquine Analogs

The anti‐inflammatory and immunomodulatory actions of chloroquine analogs have also been beneficial in the treatment of viral infections and associated pathologies (Al‐Bari 2015). Several studies have suggested that multiple organ failure and hypovolemic shock observed in fatal cases are most likely associated with not only the direct viral infection and destruction of susceptible cells (e.g., endothelial cells), but also the effects of proinflammatory cytokines, chemokines and other mediators released from infected and activated cells such as monocytes and macrophages (Yang et al. 1998; Baize et al. 2002; Marzi et al. 2012; Geisbert et al. 2015). One of the cytokines strongly implicated in filoviral pathologies is tumor necrosis factor‐*α* (TNF‐*α*) which is able to activate macrophages to release mediators such as ROS, NO and other molecules of TNF‐*α*. These cytokines cause to increase both the permeability and infectivity of endothelial cells (Tracey and Cerami 1994; Baize et al. 2002). It is suggested that therapeutic agents like chloroquine analogs which are able to prevent the activation of macrophages and inhibit the secretion of TNF‐*α* from various cells at clinically relevant concentrations (Al‐Bari 2015) would confer some benefits in the treatment of viral infections. Another cytokine, interferon‐*γ* (IFN‐*γ*) has also been implicated in the pathologies of viral infections (e.g., Ebola). It has been reported that IFN‐*γ* increase cellular sensitivity to apoptosis by up‐regulating the expression of Fas and Fas ligand (Schroder et al. 2004), in fatal case of Ebola infection (Baize et al. 1999). IFN‐*γ* also takes a part of massive apoptosis by stimulating monocytes/macrophages which produce neopterin (Baize et al. 2002) and its concomitant derivative, 7‐8‐dihydroneopterin (Murr et al. 2002). It has been shown that the plasma concentration of neopterin significantly and progressively increases throughout the disease course of Ebola infections (Baize et al. 2002). Therefore, therapeutic agents like chloroquine analogs, which inhibit various cytokine production (e.g., IFN‐*γ*, TNF‐*α*, neopterin) from various cells and also able to prevent the activation of macrophages (Baize et al. 2002; Al‐Bari 2015), these analogs may become a great therapy in the treatment of patients infected with emerging viruses.

As an adjuvant therapy, chloroquine analogs regulate immune activation in viral infection (e.g., HIV‐1) with other antiretroviral agents. The analogs reduce systemic T‐cell activation (Murray et al. 2010; Leroux‐Roels et al. 2014; Routy et al. 2014) and immune hyperactivation in HIV/AIDS (Savarino and Shytaj 2015). Thus, the analogs are beneficial for chronic HIV‐infected individuals. As an endosomal inhibitor, chloroquine blocks Toll‐like receptor (TLR) mediated activation of plasmacytoid dendritic cells (pDC), and myeloid differentiation primary response gene 88 (MyD88) signaling by the decrease in levels of the downstream signaling molecules, interleukin‐1 receptor associated kinase 4 (IRAK‐4) and IFN regulatory factor 7 (IRF‐7) and by the inhibition of IFN‐*α* synthesis (Martinson et al. 2014). In addition to suppress pDC activation, the analogs also block the negative modulators of T‐cells such as indoleamine 2,3‐dioxygenase (IDO) and programmed death ligand 1 (PDL‐1). Since TLR stimulation and production of IFN‐*α* by pDC contribute to immune activation, blocking the pathway using chloroquine analogs will interfere emerging viral pathogenesis (Martinson et al. 2014).

## Effectiveness of Chloroquine Analogs in Emerging Viruses

### HIV

The productive entry and replication of AIDS causative agent, HIV‐1 are dependent on the endocytic pathways and involve acidic organelles, such as endosomes, lysosomes and trans‐Golgi network (Daecke et al. 2005; Chauhan et al. 2014). Many studies have focused on the anti‐HIV activities of chloroquine analogs (e.g., chloroquine, hydroxychloroquine, pamaquine, plasmoquine or primaquine) against HIV (Leroux‐Roels et al. 2014; Martinson et al. 2014; Mizuguchi et al. 2015; Savarino and Shytaj 2015).

In vitro chloroquine and its analog hydroxychloroquine are endowed with broad‐spectrum anti‐HIV‐1 and HIV‐2 activity at clinically achievable concentrations (0–12.5 *μ*mol/L) (Savarino et al. 2001a). Chloroquine also inhibits HIV‐1 in post‐integrational event by affecting newly produced viral envelope glycoproteins. In vitro, chloroquine exerts an additive anti‐HIV‐1 effect in combination with other anti‐retroviral agents (e.g. zidovudine, didanosine and hydroxyurea) without cellular toxicity or apoptosis (Boelaert et al. 2001a; Savarino et al. 2004). Since chloroquine and hydroxychloroquine appear to have a similar site of action (i.e. post‐transcriptional inhibition of gp120); these drugs can be useful in combination with other anti‐retroviral agents for the treatment for HIV‐1 infected individuals in the developing world (Boelaert et al. 2001a; Savarino et al. 2001b; Naarding et al. 2007). As a HIV inhibitor, chloroquine alone inhibits HIV replication and viral particle glycosylation and synergizes the inhibitory effects with protease inhibitors such as indinavir, ritonavir, or saquinavir (Savarino et al. 2004). Thus, it is suggested the use of chloroquine analogs in the management of routine HIV disease in vivo (Romanelli et al. 2004; Parris 2006; Naarding et al. 2007). HIV‐1 transmission and replication on CD4^+^ T‐lymphocytes are reduced in presence of chloroquine, suggesting that the analogs exert anti‐HIV‐1 activity through a number of mechanisms in vivo including modulations of the gp120 structure (Naarding et al. 2007). As an inhibitor of route of entry, chloroquine vaginal gel formulation also exerts anti‐HIV‐1 activity in vitro (Brouwers et al. 2008). pDC cells recognize microbial products and viruses via TLR7 or TLR9, and produce IFNs. The presence of elevated IFN‐*α* level in HIV infected cells leads to contribute the immune activation. Chloroquine blocks TLR‐mediated activation of pDC and MyD88 signaling by decrease in the levels of the downstream signaling molecules IRAK‐4 and IRF‐7 and by inhibition of IFN‐*α* synthesis (Ewald et al. 2008; Martinson et al. 2014). Chloroquine also decreases CD8^+^ T‐cell activation induced by HIV‐1. These results suggest that chloroquine analogs have a preventive role in HIV pathogenesis by blocking TLR stimulation and IFN‐*α* production pathway (Martinson et al. 2014). Interestingly, recently in order to find out, screen and evaluate other anti‐HIV compounds such as cell‐penetrating peptides or polyfunctional styryl thiazolopyrimidines, the analogs can be used as standard drugs for comparison purposes (Fatima et al. 2012; Mizuguchi et al. 2015).

In vivo, the use of chloroquine in HIV positive mothers is associated with a decreased rate of vertical transmission of the virus to their infants (Neely et al. 2003) and children (Boelaert et al. 2001b). Chloroquine in combination with zidovudine and didanosine has a therapeutic potential for HIV‐1 infected children (Engchanil et al. 2006). Increased level of activated T cells is a hallmark of chronic HIV infection and the treatment with chloroquine reduces systemic T‐cell immune activation, suggesting that its use might be beneficial of HIV‐infected individuals (Murray et al. 2010). Malaria and HIV co‐infection are coendemic in a wide portion of the world and remain a major cause of morbidity and mortality (Uneke and Ogbonna 2009; Kraft et al. 2015). The analogs offer significant advantages to current therapy due to lack of cross‐resistance with other antiretrovirals and low cost (Romanelli et al. 2004; Savarino et al. 2004). As chloroquine analogs inhibit HIV replication and glycosylation with limited toxicity in vitro as well as the antiviral effects of the analogs in combination with other antiretrovirals have been confirmed in vivo (Table 1), the analogs have been attempted to use in several ongoing clinical trials (Table 2). Recently, one of the review papers suggests the rational uses of chloroquine analogs counteract immune activation in HIV infection (Savarino and Shytaj 2015). However, another retrospective review confronts the statement of ‘dampening of immune activation by the analog’ (Chauhan and Tikoo 2015). Thus, if the analog‐based strategies pursue in future studies, some factors must be taken into considerations such as dose‐ and dosage form selection, starting time for treatment etc., in order to maximize the effectiveness of the analogs in vivo.

**Table 1:** Outcome of clinical trials and animal studies on the efficacy of chloroquine analogs

Disease	Analog & route	Dose & Duration	Design of study	No. of subject	Therapeutic effect and outcome
HIV‐1	HCQ, PO	800 mg/day; 8 weeks	RCT	40	IL6 decline; moderate efficacy (Sperber et al., 1995)
HIV^+^mother	CQ, PO		Pilot study cohort	287	decreased HIV vertical transmission Beneficial maternal use (Neely et al. 2003)
Chronic HIV	CQ, PO	250–500 mg/day; 8 weeks	RCT	12	Reduction in T‐cell immune activation; no effect on viral load (Murray et al. 2010)
HIV	HCQ, PO	400 mg/day; 6 month	Prospective study	20	Significant reduction in immune activation; useful immunomodulant (Piconi et al., 2011)
HIV	HCQ, PO	400 mg/day, 1/daily; 48 weeks	RCT	42	Decline in CD4 cell count; increased viral load; no efficacy (Paton et al., 2012)
HIV‐1	CQ, PO	300 mg, vaccine 2 day after CQ	CT, Phase II		CD4^+^ T‐cell responses; induced robust antibody response (Leroux‐Roels et al. 2014)
HIV	CQ, PO	Weeks	CT		IFN*α*2 increased, not immune activation; not beneficial (Routy et al. 2014)
Dengue	CQ	D1, 600 mg; D2,D3 300 mg	RCT	153	Reduction in fever clearance time; no efficacy (Tricou et al., 2010)
Dengue	CQ	500 mg/day; 3 day	RCT	19	Decrease in pain intensity; improved dengue‐related symptoms (Borges et al., 2013)
HCV	CQ; IP	200 mg, 2/week	Case report	1	Gradual regression of the skin lesions,; effective (Pellicelli et al. 2012)
Chikungunya arthritis	CQ	20 weeks	Open pilot study	10	50% improved conditions; effective (Brighton 1984)
Chikungunya infections	CQ, PO	600 mg d1,2,3; 300 mg day 4,5	RCT	27	No significant decrease in viremia; poorly effective (De Lamballerie et al. 2008)
Influenza A	CQ, PO	500 mg/day 1/week, 1/1–12 weeks	RCT	724	Not effective (Paton et al. 2011)
Influenza A	CQ, PO	500 mg/day	Prospective Studies	105	Restored Influenza A vaccine response; beneficial (Borba et al. 2012)

PO, oral administration; IP, intraperitoneal; RCT, randomized, controlled clinical trial; CT, clinical trial;; m, months; CQ, chloroquine; HCQ, hydroxychloroquine.

**Table 2:** Clinical trials of chloroquine analogs as antiviral therapies

Status	Condition	Phase	Intervention	Trial ID
Terminated, has results	HIV Infections	Phase 2, 3	CQ 250 mg/500 mg, Placebo	NCT00308620
Completed	HIV Infections	Phase 2	GSK Biologicals’ HIV vaccine, CQ 300 mg	NCT00972725
Recruiting	HIV		CQ prophylaxis	NCT01650558
Completed	HIV		CQ	NCT02004314
Unknown	HIV Infections		HCQ, Placebo	NCT01067417
Recruiting	HIV Infection	Phase 1	HCQ	NCT01232660
Recruiting	Acute HIV Infection	Phase 1, 2	Vorinostat, HCQ, Maraviroc, Tenofovir, Emtricitabine, Efavirenz, Darunavir	NCT02475915
Active, not recruiting	HIV		ASA, HCQ	NCT02079077
Unknown	HIV Infections	Phase 2	HCQ, Placebo	NCT01067417
Recruiting	Malaria, HIV	Phase 3	DP	NCT02282293
Unknown	HIV, Malaria		MQ, placebo	NCT00499876
Completed	Toxoplasmosis, Cerebral; HIV		Atovaquone	NCT00001994
Not yet recruiting	Malaria, HIV	Phase 1	MQ, SP	NCT02524444
Unknown	Dengue	Phase 1, 2	CQ, Placebo	NCT00849602
Unknown	Hepatitis, Autoimmune	Phase 4	CQ 250 mg, Placebo	NCT01980745
Active, not recruiting	Autoimmune Hepatitis	Phase 4	CQ, PS, azathioprine	NCT02463331
Recruiting	Hepatitis C Virus	Phase 4	CQ 150 mg, placebo	NCT02058173
Unknown	Chronic Hepatitis C	Phase 1, 2	HCQ, Pegylated‐IFN *α*‐2a, Ribavirin	NCT01272310
Terminated, has results	Hepatitis C	Phase 1, 2	Ribavirin, HCQ	NCT01833845
Terminated	Chikungunya Virus	Phase 3	CQ	NCT00391313
Unknown	Influenza	Phase 2	CQ, Placebo	NCT01078779
Recruiting	EboV Disease	Phase 2	Favipiravir	NCT02329054
Terminated, has results	PML	Phase 1, 2	MQ	NCT00746941
Not yet recruiting	Rabies	Phase 4	CQ, Atovaquone and Proguanil, Doxycycline, Rabies vaccine	NCT02564471

CQ, chloroquine; HCQ, hydroxychloroquine; PS, prednisone; ASA, acetylsalicylic acid; IFN, interferon; DP, dihydroartemisinin‐piperaquine; MQ, mefloquine; SP, sulphadoxine‐Pyrimethamine; HIV, human immunodeficiency virus; PML, progressive multifocal leukoencephalopathy.

### Dengue and hepatitis C viruses

Flaviviruses, including hepatitis C virus (HCV) and dengue virus, affect millions of people worldwide, and an estimated 3.2 million people in the United States. These viruses display a broad spectrum of clinical manifestations which may vary from asymptomatic to severe and even fatal features (Dedania and Wu 2015). The dengue viral envelope glycoprotein E, and HCV glycoproteins E1 and E2 play important roles for their attachments and entries into cells through two main pathways: direct fusion at the plasma membrane and receptor‐mediated endocytosis. The fusion process of these viruses is facilitated by low pH within the endosome (Peng et al. 2009; Matsuda et al. 2014; Piccini et al. 2015). Once exposed to acidic pH containing endosomal vesicles of the target cells, the viruses rearrange their structural conformational modifications of envelope glycoproteins (e.g., trimerization of dengue glycoprotein E) and finally initiate their replications (Ashfaq et al. 2011; Gandini et al. 2013; Vausselin et al. 2013; Bekerman and Einav 2015).

By increasing endosomal pH, chloroquine inhibits dengue virus type 2 replication (DENV‐2)in Vero cells (Farias et al. 2013), and U937 cells (Farias et al. 2014) at a nontoxic dose of 50 *μ*g/mL in vitro. Amodiaquine, one of the 4‐aminoquinoline drugs inhibits DENV‐2 replication and infectivity with EC_90_ value 2.69 *μ*mol/L in the replicon expressing cells (Boonyasuppayakorn et al. 2014). Chloroquine also inhibits DENV‐2‐induced membrane TNF‐related apoptosis‐inducing ligand (mTRAIL) relocalization and IFN‐*α* production by pDCs in vitro and in vivo (Gandini et al. 2013). Chloroquine interferes in DENV‐2 replication in Aotus monkeys. The serum concentrations of TNF*α* and IFN*γ* are statistically significant reduced in chloroquine treated groups (Farias et al. 2015). Several analogs of chloroquine have been attempted to use in several randomized, double‐blind studies and the outcomes of the therapies are shown in Tables 1, 2.

Chloroquine analogs also reduce HCV entry, replication and infection by interfering endosomal acidification. Treatment of different cells such as JFH‐1 or Huh‐7 with chloroquine suppresses entry and replication of HCV in a dose‐dependent manner (Blanchard et al. 2006; Mizui et al. 2009). Chloroquine shows more than 50% reduction in infectivity of HCV at 50 *μ*mol/L concentrations in liver cells (Ashfaq et al. 2011). Furthermore, combination treatment of chloroquine to IFN*α* enhanced the antiviral effect of IFN*α* and prevents re‐propagation of HCV (Mizui et al. 2009). Ferroquine, a ferrocenic analog of chloroquine, potently inhibits HCV infection of hepatoma cell lines by affecting an early step of the viral life cycle. In addition, the analog also inhibits HCV RNA replication, and impairs the fusion process. The analog also suppresses HCV cell‐to‐cell spread between neighboring cells. Combinations of the analog with IFN, or an inhibitor of HCV NS3/4A protease, result in additive to synergistic activity (Vausselin et al. 2013). By reducing acidification of endocytic system, chloroquine enhances human CD8^+^ T cell responses against soluble viral antigens (derived from HCV, hepatitis B virus, or HIV) in vivo (Accapezzato et al. 2005). A case report suggests that a patient with HCV infection is associated with porphyria cutanea tarda and chloroquine (200 mg twice weekly) results in a gradual regression of the skin lesions including porphyria (Pellicelli et al. 2012).

### Chikungunya viruses

Chikungunya virus (CHIKV) is a mosquito‐transmitted alphavirus that causes an acute fever characterized with long‐lasting arthralgia, affecting primarily the peripheral joints in humans (Sourisseau et al. 2007; Taubitz et al. 2007). The mechanisms of CHIKV entry and infection into the host cells indicate that CHIKV is endocyted into *Aedes albopictus* cell lines and requires a low pH‐dependent viral membrane fusion process (Ozden et al. 2008; Gay et al. 2012).

Treatment strategies of CHIKV infection are primarily supportive and symptomatic. Although there is no generally recommended specific antiviral therapy, the use of chloroquine, ribavirin and interferon‐alpha might be useful (Stock 2009). A milestone in the fortunes of chloroquine analogs is occurred since 1984 through an open pilot study on the efficacy of chloroquine in the treatment of chronic CHIKV arthritis and the observations indicated that antimalarial treatment improved significantly the articular index and morning stiffness in patients with CHIKV infection (about 50% patients improve their conditions) (Brighton 1984). This result leads to justify further studies of chloroquine in CHIKV infection. In vitro range, chloroquine analog shows a potential antiviral activity against CHIKV (Kaur and Chu 2013). Inhibitory effects are observed when chloroquine is administered preinfection, postinfection, and concurrent with infection, suggesting that chloroquine has prophylactic and therapeutic potential. Chloroquine diminishes CHIKV infection in a dose‐dependent manner (range of effective concentration, 5–20 *μ*mol/L). The maximum inhibitory effect is observed within 1–3 h postinfection, and treatment is ineffective when the virus successfully passes through the acidification pathway in early stages of infection (Stock 2009; Kaur and Chu 2013). Thus, the starting time of treatment, doses and dose regimens of chloroquine are important considerations for successful treatments of CHIKV infections. In vivo, chloroquine is not or poorly active against acute CHIKV infections (De Lamballerie et al. 2008, 2009). This may be proper prognosis of the infection and the initial time of treatment, as well as dose regimens of chloroquine has not been established at yet.

### Influenza A and Newcastle disease viruses

Orthomyxovirus influenza A virus (IAV) represents a major threat to human health. IAV enters into host cells through clathrin‐dependent endocytosis (Di Trani et al. 2007; Wang and Jiang 2009). The influenza viral surface glycoprotein, hemagglutinin (HA) also facilitates the viral entry into host cells by mediating the fusion of viral membrane within cellular acidic compartments (Wu et al. 2015). Thus, intravesicular acidic pH is essential for the viral‐cell fusion process.

In vitro, chloroquine is able to inhibit IAV replication at lower plasma concentration than that reached during treatment of acute malaria (Ooi et al. 2006). Chloroquine increases endosomal pH and impairs IAV release into the cytosol (Fedson 2008). The inhibitory effect of chloroquine is maximal when the drug has been given at the time of infection and is lost after 2 h postinfection (Di Trani et al. 2007). These results suggest that the treatment timing approximately corresponds to that of virus/cell fusion. Moreover, there is a clear correlation between the EC_50_ of chloroquine in vitro and the electrostatic potential of HA mediating the virus/cell fusion process. Thus, treatment should be started within time of virus/cell fusion process with exact effective concentration.

Although in vitro results are promising, chloroquine is not effective as preventive therapy in vivo in standard mouse and ferret models of human IAV infection (Vigerust and Mccullers 2007). Recently, chloroquine is highly effective in treating avian IAV infection in an animal model (Yan et al. 2012). Chloroquine improves CD8^+^ T cell responses in mice following a single administration of influenza vaccines (Garulli et al. 2013). Although the analogs have been reported to be effective against IAV in vitro and used in in‐vivo experiments and clinical trial for prevention or treatment of influenza (Paton et al. 2011; Borba et al. 2012), the effectiveness of analogs as anti‐influenza drugs is questioned, and cautions in their uses are recommended (Wu et al. 2015).

Newcastle disease virus (NDV) is the causative agent of veterinary diseases (birds). NDV enters the cell by direct fusion of the viral envelope with the cellular membrane and by low pH‐ and receptor‐dependent endocytosis (Sanchez‐Felipe et al. 2013). In vitro, optimal NDV infection of the host cells is significantly affected by drugs such as chloroquine that inhibit endosomal acidification (Sanchez‐Felipe et al. 2013). NDV infection induces autophagy and inhibition of autophagy by the analog reduces the viral replication and infection (Meng et al. 2012; Sun et al. 2013). Importantly, as a pharmacological modulator of autophagy, chloroquine potentiates NDV‐mediated oncolysis in mice bearing cisplatin‐resistant lung cancer cells (Jiang et al. 2014).

### Ebola and marburg viruses

Filoviruses (Ebola and Marburg) cause severe hemorrhagic fever in humans and nonhuman primates. The peplomers of Ebola viruses are composed of trimerized heterodimers of glycoproteins 1 and 2, which are heavily glycosylated with both N‐linked and O‐linked glycans (Geisbert et al. 2015). The glycoproteins of Ebola peplomers have broad tropism for a variety of host cells due to their ability to bind either specifically or non‐specifically to various cell surface molecules and facilitate the pH‐dependent endosomal entry to the host cells (Yang et al. 1998, 2000; Bhattacharyya et al. 2010). Thus, despite this broad tropism, infection by filoviruses greatly depends on acidic pH (Chandran et al. 2005; Marzi et al. 2012). Using in vitro cell culture assays, a systematic screening of FDA‐approved drugs for inhibitors of biological threat agents such as Ebola and Marburg viruses has been performed and found that chloroquine is the most noteworthy antiviral compound among the identified multiple virus‐specific inhibitors. In this report, it has been suggested that chloroquine disrupts viral entry and replication in vitro; protects mice against Ebola virus challenge in vivo (Madrid et al. 2013). Long 2015 (Long et al. 2015) confirmed that chloroquine is capable to inhibit viral entry in a pH specific manner and considered it as a priority candidate for treatment of Ebola viruses. Later on, reports suggested chloroquine inhibits the virus replication in vitro but is unable to treat in patient (Bishop 2014); mouse, hamster (Falzarano et al., 2015) and guinea pigs (Dowall et al. 2015).

### SARS and MERS viruses

The SARS coronavirus (CoV) recognized in 2003 has infected about 8000 people worldwide, with a fatality rate of approximately 10%. The infection of target cells by the SARS‐CoV is mediated through the interaction of the viral spike protein and its cellular receptor, angiotensin‐converting enzyme 2 (ACE2) (Freund et al. 2015). MERS coronavirus (CoV) infection occurs in the same phenomena of SARS in humans, with the exception of cellular entry receptor, dipeptidyl peptidase 4 (DPP4) (Li et al. 2003; Raj et al. 2013).

Registered effective prophylactics or postexposure therapeutics for the treatment of coronaviral infections are not currently available. It has been reported that chloroquine has strong antiviral effects on SARS‐CoV infection and spread in vitro (Keyaerts et al. 2004; Vincent et al. 2005; De Wilde et al. 2014). In addition to the well‐known functions of chloroquine such as elevations of endosomal pH, the drug appears to interfere with terminal glycosylation of the cellular receptor, ACE2. This may negatively affect the virus‐receptor binding and abrogate the infection. The IC_50_ of chloroquine for inhibition of SARS‐CoV in vitro (8.8 ± 1.2 *μ*mol/L) is significantly lower than its cytostatic activity which approximates the plasma chloroquine concentrations reached during treatment of acute malaria. More interestingly, the suppressing effect is observed when the cells are treated with chloroquine either before or after exposure to the virus, suggesting both prophylactic and therapeutic advantage (Keyaerts et al. 2004; Vincent et al. 2005). There are screened a library of 348 FDA‐approved drugs for anti‐MERS‐CoV activity in cell culture and only four compounds (chloroquine, chlorpromazine, loperamide, and lopinavir) have been identified to inhibit the viral replication (50% effective concentrations, EC_50_ 3–8 *μ*mol/L). Although the protective activity of chloroquine (alone or in combination) remains to be assessed in animal models, these findings may offer a starting point for treatment of patients infected with zoonotic coronaviruses like MERS‐CoV (De Wilde et al. 2014).

### Other viruses

Betanodaviruses are the causal agent of viral nervous necrosis in many species of marine farmed fish. It is reported that betanodaviruses enter fish cells by endocytosis and the presence of 1 *μ*mol/L chloroquine inhibits the entry and infection of betanodaviruses in vitro (Adachi et al. 2007). Feline calicivirus (FCV) is a major causative agent of respiratory disease in cats. FCV infects cells via clathrin‐mediated endocytosis. Inhibitors of endosome acidification such as chloroquine block the viral permeabilization event in endosome (Stuart and Brown 2006). Treatment with chloroquine significantly reduces the replication of caliciviruses including porcine enteric calicivirus, murine norovirus‐1 and feline calicivirus in vitro (Shivanna et al. 2014). Borna disease virus (BDV), mainly seen as the causative agent of borna disease in horses and other animals, exhibits high neurotropism and provides an important experimental model system for studying virus‐cell interactions within the central nervous system. Lysosomotropic agents including chloroquine prevent BDV infection, indicating that BDV enters host cells by endocytosis and requires an acidic intracellular compartment to allow membrane fusion and initiate infection (Gonzalez‐Dunia et al. 1998; Clemente and De La Torre 2009).

### Enigma of clandestine association with failure of chloroquine analogs clinically

Chloroquine analogs have become potential candidates for treatment of several emerging viral diseases on the basis of their inhibitory effects in vitro. Although these effects proved highly reproducible, the in vivo antiviral effects of the analogs have been proved in limited extents. Several following reasons exist for the failure of the analogs to become potential antiretroviral agents in clinically:

1. Chloroquine and its analogs such as hydroxychloroquine are ineffectiveness in treating low pH‐dependent emerging viral infections due to failed to attain and sustain steady state concentrations in blood sufficient to increase and keep the pH of the acidic organelles to approximately neutral until patients’ viremia becomes undetectable. Although there is a considerable intersubject variability in the steady state blood concentrations of chloroquine analogs, the maximum safe serum concentration of chloroquine diphosphate is 250–280 ng/mL at maximum safe dose of 4 mg/kg per day (Laaksonen et al. 1974) and whole blood concentration of hydroxychloroquine is 1.0–2.6 *μ*g/L (Munster et al. 2002). However, the doses and dose regimens should be adjusted to optimize the benefit/risk ratio on the rational basis of pharmacokinetics and pharmaco/toxicodynamics considerations. Moreover, the plasma levels of chloroquine analogs depend on some other factors such as methodology as well as storage conditions of samples because the analogs have been trapped in erythrocytes, lymphocytes and platelets. Chloroquine is also a racemic mixture. It has been reported that the kinetic behavior of separate enantiomers differs in humans (Augustijns et al. 1992). There is nothing to know about the relevance of stereospecificity in the therapy of emerging viral infections.
2. For pharmacokinetic parameter considerations, chloroquine has been disputed for its narrow therapeutic indexes and poor penetration in specific tissues (Augustijns et al. 1992). It is reported that there are differences in efficacy and toxicity between chloroquine and its analog, hydroxychloroquine for long‐term effectiveness in rheumatoid diseases (Avina‐Zubieta et al. 1998). In rheumatoid arthritis therapy, it is reported that hydroxychloroquine is one half to two‐thirds as effective as chloroquine but one half in the toxicity (Tobin et al. 1982; Mackenzie 1983). Thus, hydroxychloroquine is generally regarded as a safe, reasonably effective and less toxic for the treatment of rheumatoid diseases, with a recommended daily dose of <6.4 mg/kg per day and a maximum dose of 400 mg/day. It is suggested that the blood concentration of desethylhydroxychloroquine, one of the oxidative metabolites of hydroxychloroquine is related to treatment efficacy, and that the blood hydroxychloroquine concentration is associated with toxicity (Munster et al. 2002). Thus, selection of chloroquine analogs or its metabolite is also an important factor for successful treatment of viral diseases. Chloroquine analog in combination with other antiviral drugs is considered for effective treatment of the viral diseases in order to avoid the interaction of P‐glycoprotein and multidrug‐resistance associated proteins of these viruses, which extrude drugs from the cells and other anatomic compartments (Savarino 2011). It is also noted that the combined drugs must not be interacted with the analogs (Zhou et al. 1995).
3. The maximum inhibitory effect of chloroquine analogs is observed immediately started and treatment is ineffective when the virus successfully passes through the replication events (Stock 2009; Kaur and Chu 2013). It is also noted that the efficacy of chloroquine analog is markedly dependent on the acute stage and severity of infections (De Lamballerie et al. 2008). Thus, the starting time of treatment, doses and dose regimens (therapeutic loading dose and subsequent maintenance dose to achieve steady state blood chloroquine concentration) are important factors for efficacy in these viral infections.

It is hope that the exact chloroquine analog with improved pharmacokinetics will be able to bridge the gap of effectiveness between the in‐vitro and in‐vivo effects in the future.

## Conclusions and Future Perspective

Since inhibition of acidification of endosomes during replication courses of the emerging viruses by chloroquine analogs have been reported to be endowed with a wide range of viral diseases including HIV, the following consequence can be taken into considerations (1) Chloroquine inhibits viral entry, replication and infection. (2) The analogs exert an inhibitory effect on several opportunistic pathogens including viruses (in AIDS). (3) The analogs exert an inhibitory effect on the synthesis of several pro‐inflammatory cytokines that may play a pathogenic role in the progression of viral infection. (4) The drugs have the potential to restrict iron accumulation in various tissues that may play a negative role in viral infection. (5) The analogs have practical advantages, as they are widely distributed, inexpensive and not stigmatizing. (6) The analogs may be of potential benefit in decreasing the rate of mother‐to‐child transmission of viruses like HIV.

Based on the results of various in vitro studies suggest that chloroquine analogs ameliorate in infections from low pH dependent viruses. Although the outcomes of the clinical trials are not impressive (Table 1) the analogs had some potential in clinical efficacy to viral infections. Several clinical trials (still ongoing) have attempted to establish the use chloroquine analogs in the prevention or treatment of several viral infections, including HIV, hepatitis, rabies, Chikungunya, Ebola virus disease, influenza A and B, and dengue viral infections (Table 2).

In this review, it is tried to establish a bridge the gap between the promising in vitro results of chloroquine analogs as broad spectrum antivirals and the mixed or disappointing results obtained in several animal models and clinical trials. There are many factors to get a feasible solution to bridge this gap for exerting the antiviral effects such as sustained release dosage form modification to maintain continuous steady state levels of chloroquine analogs, selection of suitable analog combination therapy or preventive dosage form (e.g. vaginal gels for anti‐HIV).

Recently, a number of tracks for suitable chloroquine analogs are under investigation (inhibition of viral enzymes, of virus entry or maturation, enhancement of immunological response) and new animal models have been made available, including a mouse model and a nonhuman primate model for development of better therapy for viral diseases. Along with, computational models might be helpful for docking and targeting the molecular features which are responsible for antiviral activities of chloroquine analogs (e.g., targeting VP35 of Ebola viruses by the analog) (Ekins et al. 2015) and the hypotheses can be used therapeutically as well as in clinical trials. Much more double‐blind placebo‐controlled randomized trials are necessary for justification the use of chloroquine analogs in next coming emerging viral treatment.

## Disclosure

The author wish to confirm that there are no known conflicts of interest associated with this publication and there has been no financial support for this work that could have influenced its outcome. The author also declared not to receive any assistance of a professional medical writer or similar service. Thus, it can be stated ‘None to declare’.
